# Is Europe Running out of Chemistry Teachers?

**DOI:** 10.1021/acscentsci.5c00038

**Published:** 2025-01-15

**Authors:** Vanessa Zainzinger

Last year, the Royal Society of Chemistry (RSC) wrote to the UK Parliament with a pressing concern. The society’s latest research had found that 30% of the country’s state secondary schools do not have enough chemistry teachers. In addition, the number of students entering chemistry undergraduate courses had been declining year after year.

**Figure:**
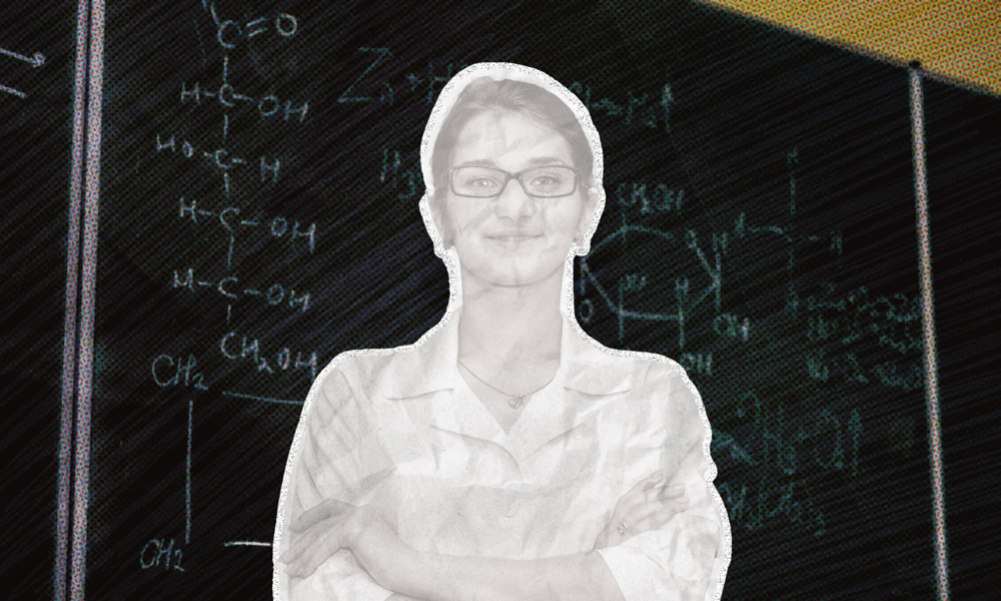
Credit: Madeline Monroe/C&EN/Shutterstock

A vicious circle was developing: fewer chemistry graduates meant fewer chemistry teachers to inspire future generations of scientists. If the government didn’t address the lack of teachers, the RSC argued, young people could be kept from pursuing a career in chemistry.

Teacher shortages are a known problem in the UK, where poor recruitment and retention rates have plagued schools for more than a decade. Increasingly, the teacher drought is spreading to the continent, affecting countries like Sweden and Germany. A recent European Commission report found teacher shortages in almost all countries in the European Union.

Across all countries, finding teachers for science, technology, engineering, and mathematics (STEM) subjects presents a high hurdle, and chemistry is among the hardest to recruit for.

“Chemistry has always been on the side of struggling,” says Jack Worth, the school workforce lead at England’s National Foundation for Education Research (NFER), which has been tracking teacher recruitment and retention rates in England since 2019. Among the data it collects are figures on teacher recruitment targets set by the UK Department for Education each year. In all the years of NFER tracking, chemistry has never met its target.

**Figure:**
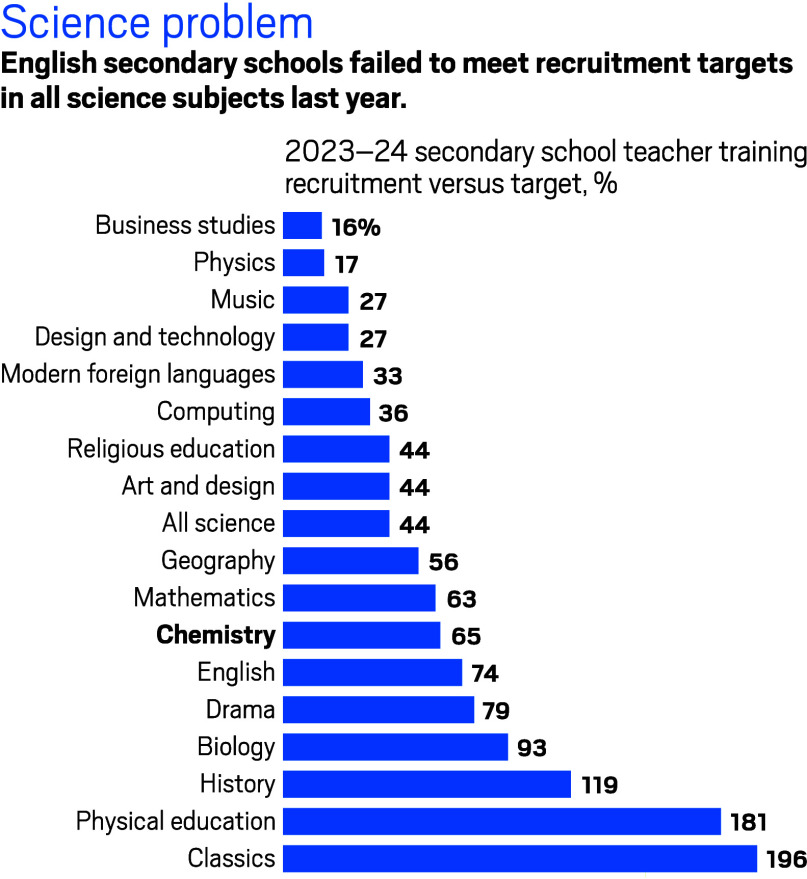
Source: UK’s Department for Education.

“There was a time during the [COVID-19] pandemic when chemistry almost met the government recruitment target because more people were applying for teaching jobs during that time,” Worth recalls. “But since then, it’s dropped again.”

In the 2023–24 school year, schools in England were able to recruit only 65% of the teaching staff they needed for chemistry, according to NFER research based on UK Department for Education data. That’s a lot better than staff recruitment for physics, which reached only 17% of its target, but worse than biology—an outlier among science subjects—for which schools recruited 93% of the teachers needed.

Young chemists have many reasons to avoid a teaching career. The most obvious one, Worth says, is that chemistry graduates can earn more money in the chemical industry. On average in the UK, entry-level chemistry teaching positions start at £32,500 ($42,000) per year. That matches the average starting salary in the chemical industry, but a gap between career paths forms after a few years.

“The starting salary might be relatively attractive, but teachers reach the top of the scale after about 5 years,” says Andy Harvey, national officer for education at the Educational Institute of Scotland (EIS), a union that represents educators in Scotland. Young teachers soon find themselves without opportunities for promotion, Harvey says. “The thing with chemistry teachers is, they do have marketable skills. They can get better pay elsewhere.”

Unions like the EIS are unimpressed by some widely publicized UK government recruitment schemes, such as grants of up to £28,000 ($36,000) for those enrolling in chemistry teacher training courses and an extensive “Get into Teaching” media campaign. These efforts might lure people to the classroom, but they won’t make them stay, Harvey says. Retention rates are terrible. EIS’s most recent school survey found that 40% of chemistry teachers working in Scotland are considering leaving the profession within the next 5 years.

It’s not only about the money. Workload, stress, and anxiety are also reasons why teachers decide to throw in the towel, says Annette Farrell, the RSC’s program manager for education policy. The RSC’s annual science teacher survey—which covers school science technicians as well as chemistry, physics, and biology teachers across the UK—found that half of those planning to leave their job will do it because they feel exhausted and underappreciated.

“A lot of people also say student behavior has become more challenging since the pandemic, and this seems to be a contributing factor when it comes to wanting to leave,” she says.

Farrell, who taught biology before joining the RSC, connects worsening student behavior to a lack of school funding, which has resulted in bigger class sizes and fewer teaching assistants to help look after pupils.

With low retention rates, unmet recruitment targets, and a student population expected to rise by 29,000 between 2024 and 2028, chemistry teaching in the UK is in a downward spiral. And the issue crosses borders. The European Commission’s most recent Education and Training Monitor report found that 24 of the 27 European Union member states are affected by STEM teacher shortages. Only Croatia, Cyprus, and Greece did not report a lack of education staff. The data do not break out chemistry teachers.

Sweden is one of the worst affected. It needs 153,000 qualified teachers overall by 2035 and has little hope of meeting this target. “It is worrying and has been for a long time,” says Lee Gleichmann, director of education for the Swedish National Agency for Education. In Sweden, teachers work nearly 5 h per week more than their European peers, and only 10.7% of teachers believe their profession is valued by society.

“Young people in Sweden who are about to choose a career path have many options, and the teaching profession may not be top of mind for everyone,” Gleichmann says.

Gleichmann says teaching has an image problem. Few would choose to enter a career that offers limited pay for a high workload, inflexible work hours, and little social status. “The image of the school in the media and social media also needs to change,” she says.

Teaching enjoys a higher status in Germany, where teachers at public schools are civil servants. The connected benefits—such as lower tax rates and a high level of job security—generally keep teachers from quitting, says Gesa Bruno-Latocha from the German Education Union. “If someone decides to leave the job nonetheless, then they must be in a really bad place,” she says.

And yet studies expect Germany to fall short by up to 80,000 teachers by 2035, while the number of pupils is due to increase by 9.8%. Numbers on chemistry teachers specifically are available for only one German state, North Rhine-Westphalia. There, only a third of chemistry teaching positions will be filled in 2030, according to a 2020 study that Bruno-Latocha says is still the most reliable predictor of STEM teacher numbers in Germany.

The imminent shortage is due partly to an upcoming retirement wave, Bruno-Latocha says. The latest numbers released by the German Federal Statistical Office found that a third of teachers in Germany are older than 50. Notably, eastern Germany relies on an older workforce because it suffered a tremendous drop in birthrates and a mass exodus of educated young people after the fall of the Berlin Wall in 1989. It will be losing a huge chunk of its teaching workforce in one fell swoop.

German states, which take individual responsibility for their own education systems, are now trying to counter the retirement wave by luring young chemists into teaching. “Universities and research organizations often employ scientists on fixed-term contracts, and the odd chemist gets fed up and thinks, I’d rather teach on a secure contract,” Bruno-Latocha says. State governments are offering programs that pay scientists to gain the teaching degree required to work in German schools. But so far, there are no data on how well these programs are working, she says.

**Figure:**
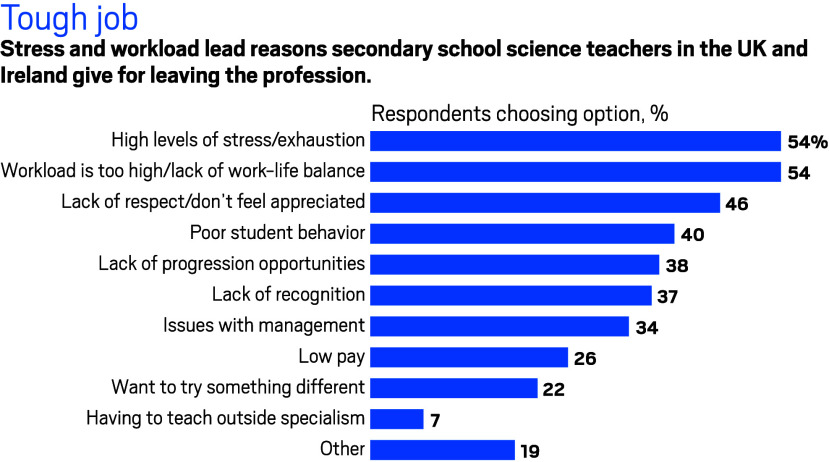
Note: Respondents were able to provide multiple reasons. Source: Royal Society of Chemistry 2023 science teaching survey.

The jury is also out in neighboring Austria, which is implementing similar programs. The Association of the Austrian Chemical Industry (FCIO) supports the schemes. It is keen on getting trained chemists into schools to prevent a solution that’s currently popular in countries including Austria and the UK: asking teachers who specialize in other subjects to cover chemistry lessons.

“Ideally, these are biology teachers who have come into contact with chemistry at least once during their training. But there are no legal regulations for this, so sometimes it also affects German or history teachers who have little connection to chemistry,” says FCIO chairman Hubert Culik.

Such nonspecialized teachers aren’t usually given additional training, and they are rarely qualified to carry out experiments in the classroom. Culik fears the dry, dispassionate lessons that typically result are a death sentence for any budding chemist’s interest in the subject. “We know that experimental teaching is what sparks interest in chemistry,” he says.

The result could be a general public that is more skeptical of the chemical industry overall, Culik fears. “If something is foreign to people, they reject it. If you got to know chemistry at school as an exciting science with problem-solving skills, then you will not be skeptical of the industry or the products from the chemical industry.”

Germany’s chemical industry trade body, the German Chemical Industry Association (VCI), is more concerned with keeping laboratory benches full. The number of students enrolling in chemistry courses at German universities has been steadily decreasing in recent years. Until 2015, well over 11,000 students enrolled every year. By 2023, the number had dropped to 8,000.

Verena Weidmann, VCI’s manager for education policy, links the drop to a lack of engaging teaching at schools. “We already have a shortage of skilled workers in the chemical industry,” she says. “Now young people’s interest in STEM subjects is decreasing, and as a result, fewer training courses and [undergraduate] studies are being started.”

Weidmann says science teachers must be given the means to keep experiment-based teaching in the classroom. This means making sure any nonchemists covering the subject get the help and education they need to teach beyond the textbook.

In the UK, the RSC is lobbying for high-quality professional development programs for teachers so they can keep up to date with changes in their subject and gain the expertise needed to effectively teach other school science disciplines. Governments and school leaders have to recognize that subject-specific knowledge is vital to inspire young people, says Farrell, who, when she was a biology teacher, was called upon to teach chemistry and physics.

“I’m sure I was just one step ahead of my students in the textbook,” she recalls. “There wasn’t anything in place to support me in building my knowledge in chemistry.”

Farrell argues that such upskilling is part of the solution to the teacher shortage: recruitment and retention rates won’t be fixed in a hurry, and for now the likes of biology teachers will be asked to plug growing gaps in other STEM subjects.

In the longer term, the main challenge will be improving recruitment and retention rates at the same time. Government schemes to fill teacher training programs will be worthless if exhausted young teachers leave the profession after a few years. Solving the issue for good means making teaching an attractive profession in terms of pay, status, and the day-to-day challenges of the job, Farrell says. This means more funding for schools, better support for struggling teachers, and more emphasis on a work-life balance that can hold its own against that of other careers in the sciences.

While there is no easy solution for such a multifaceted problem, Farrell is hopeful that teaching can be made appealing again. “There are some incredibly rewarding aspects of teaching,” she says. “It should be something that a lot of people want to do.”

## Vanessa Zainzinger is a freelance contributor to

Chemical & Engineering News, *the independent news outlet of the American Chemical Society.*A version of this story appeared in C&EN.

